# A Novel Complementation Assay for Quick and Specific Screen of Genes Encoding Glycerol-3-Phosphate Acyltransferases

**DOI:** 10.3389/fpls.2018.00353

**Published:** 2018-03-19

**Authors:** Jie Lei, Yingchun Miao, Yu Lan, Xiuxiu Han, Hongbo Liu, Yi Gan, Leilei Niu, Yanyan Wang, Zhifu Zheng

**Affiliations:** ^1^School of Agriculture and Food Science, Zhejiang A & F University, Hangzhou, China; ^2^School of Forestry and Biotechnology, Zhejiang A & F University, Hangzhou, China

**Keywords:** glycerolipid, phosphatidic acid, glycerol-3-phosphate acyltransferase, yeast complementation, heterologous expression, *Arabidopsis*

## Abstract

The initial step in glycerolipid biosynthesis, especially in diverse allopolyploid crop species, is poorly understood, mainly due to the lack of an effective and convenient method for functional characterization of genes encoding glycerol-3-phosphate acyltransferases (GPATs) catalyzing this reaction. Here we present a novel complementation assay for quick and specific characterization of GPAT-encoding genes. Its key design involves rational construction of yeast conditional lethal *gat1*Δ*gat2*Δ double mutant bearing the heterologous *Arabidopsis AtGPAT1* gene whose leaky expression under repressed conditions does not support any non-specific growth, thereby circumventing the false positive problem encountered with the system based on the *gat1*Δ*gat2*Δ mutant harboring the native episomal *GAT1* gene whose leaky expression appears to be sufficient for generating enough GPAT activities for the non-specific restoration of the mutant growth. A complementation assay developed based on this novel mutant enables quick phenotypic screen of GPAT sequences. A high degree of specificity of our assay was exemplified by its ability to differentiate effectively GPAT-encoding genes from those of other fatty acyltransferases and lipid-related sequences. Using this assay, we show that *Arabidopsis* AtGPAT1, AtGPAT5, and AtGPAT7 can complement the phosphatidate biosynthetic defect in the double mutants. Collectively, our assay provides a powerful tool for rapid screening, validation and optimization of GPAT sequences, aiding future engineering of the initial step of the triacylglycerol biosynthesis in oilseeds.

## Introduction

Glycerol-3-phosphate acyltransferase (GPAT) catalyzes the initial committed step of *de novo* biosynthesis of phosphatidic acid (PA) as a general precursor for membrane glycerolipids and the seed storage lipid triacylglycerol (TAG). Its activities have been shown to be present in the plastids, mitochondria, and endoplasmic reticulum in plant cells and extensive exchanges of glycerolipid molecules between these intracellular compartments have been established, thus underlining the complexity of glycerolipid metabolism in plant cells (Browse et al., 1986; Kunst et al., 1988; Li et al., 2015). In the last two decades, significant advance in our understanding of plastidial soluble GPAT in plants has been made (Nishida et al., 1993; Murata and Tasaka, 1997). In addition, *Arabidopsis GPAT9* gene was recently demonstrated to encode an endoplasmic reticulum-based *sn*-1 GPAT that has a role in seed TAG synthesis (Gidda et al., 2009; Singer et al., 2016; Shockey et al., 2016). Nevertheless, the question about the number of GPAT genes in *Arabidopsis* and diverse crop species remains unanswered.

With continued progress in large-scale genome sequencing efforts, a large number of sequences in diverse eukaryotic species are annotated as genes coding for glycerol acyltransferases based on the previously identified four conserved acyltransferase motifs (Lewin et al., 1999). However, given that most of membrane-bound GPATs identified so far share these conserved domains with lysophosphatidic acid acyltransferases (LPATs) catalyzing the second step of *de novo* glycerolipid synthesis (Lewin et al., 1999; Zheng et al., 2003; Kim et al., 2005), it is highly challenging to use bioinformatic tools to distinguish which type of acyltransferase each of those annotated acyltransferase sequences codes for. Also, it has remained a daunting task to predict the functional difference between different variants of homoeologous, homologous and paralogous acyltransferase sequences naturally occurring in diverse allopolyploid plant species, thereby hampering our understanding of functional redundancy and divergence within the glycerol acyltransferase gene family and further hindering a full understanding of the heterosis phenomenon occurring in polyploidy species relative to closely related diploids (Kaur et al., 2012; Renny-Byfield and Wendel, 2014). Furthermore, it remains unknown if there exist novel GPATs lacking the conserved acyltransferase domains in higher eukaryotes. As a result, genetic or chemogenetic manipulation of the TAG biosynthetic capacity as a key trait in crops has remained a big challenge. To accelerate our understanding of glycerolipid biosynthesis, we intended to develop a convenient method for functional screen of GPAT genes.

At present, the interrogation of GPAT activities is typically accomplished through conducting an *in vitro* enzymatic assay involving the use of radiolabeled glycerol-3-phosphate (Zheng and Zou, 2001). Such assay is inconvenient and unsuitable for large-scale characterization of GPAT sequences. An additional assay is based on *in vivo* complementation of glycerol auxotrophy in the *plsB* mutant of *Escherichia coli* (BB26-36) defective in GPAT activity, which has proven useful in functional analysis of GPATs of prokaryotic origin (McIntyre et al., 1977; Lewin et al., 1999; Lindner et al., 2014). However, its limited applicability has been suggested based on accumulating evidence documenting the failure of GPATs of eukaryotic origin to rescue the growth defect, likely because the eukaryotic enzymes rely upon an acyl-CoA derivative for catalysis rather than the prokaryotic acyl-ACP derivative, which is the primary acyl donor formed in the bacterial type II fatty acid synthetic pathway (Murata and Tasaka, 1997; Zhang and Rock, 2008; Mañas-Fernández et al., 2010; Lindner et al., 2014).

Owing to the presence of a certain degree of conservation in PA synthesis between lower and higher eukaryotes, yeast genetic complementation could be an alternative tool for dissecting GPAT sequences of eukaryotic origin (Rose and Broach, 1991; Athenstaedt and Daum, 1999; Zheng and Zou, 2001; Zheng et al., 2003; Wendel et al., 2009). The previous discovery that *Saccharomyces cerevisiae* harbors only two major acyltransferases (Gat1p and Gat2p) catalyzing the initial step of glycerolipid biosynthesis (Zheng and Zou, 2001; Zaremberg and McMaster, 2002) has led to the development of the conditional lethal *gat1*Δ*gat2*Δ double mutant CMY228 (*gat1*Δ*gat2*Δ+[*pGAL1::GAT1 URA3*]) in the W303 genetic background (Zaremberg and McMaster, 2002). This strain has been used for the characterization of an endoplasmic reticulum-based GPAT from *Plasmodium falciparum* (Santiago et al., 2004). In our laboratory, one additional double mutant in the genetic background of BY4742 (a strain used in the Euroscarf deletion project) has been constructed, namely NIU8 (*BY7472, gat1*Δ*gat2*Δ+[*pGAL1::GAT1 URA3*]). Like CMY228, NIU8 is fully reliant on the episomal *GAT1* gene for its survival and growth. However, one caveat associated with heterologous complementation in the *gat1*Δ*gat2*Δ double mutant bearing the native episomal *GAT1* gene is the potential false-positive screens of target genes because its leaky expression under repressed conditions may still be sufficient to generate enough GPAT activities to support the mutant growth due to its intrinsic capacity in the native cell environment (Bratschi et al., 2009). This is indeed the case for the NIU mutant in our studies.

In most cases, the catalytic activity of an enzyme is most highly expressed in its native cell environment but lower in a heterologous cell system. Accordingly, when a heterologous GPAT protein is expressed in yeast at approximately equal levels to its yeast counterpart, the former may have much lower GPAT activities (Zheng and Zou, 2001; Zheng et al., 2003). Thus, it was hypothesized that one way to resolve the false positive problem was to replace the *GAT1* episome with an episome of heterologous gene whose leaky expression under repression conditions cannot yield sufficient GPAT activities to rescue the growth defect of a conditional lethal mutant. Based on this assumption, we constructed a novel yeast *gat1*Δ*gat2*Δ double mutant bearing heterologous *AtGPAT1* gene, a member of *Arabidopsis AtGPAT* gene family (Zheng et al., 2003). The complementation assay based on this mutant was demonstrated to possess a high degree of specificity for GPAT enzyme and circumvent the false-positive screens occurring in the NIU8 strain. Using it to test the *AtGPAT* gene family, we show that distinguished from other members, *AtGPAT1, AtGPAT5*, and *AtGPAT7* possess the capacity to rescue the growth defect of the *gat1*Δ*gat2*Δ double mutant, suggesting a role for the respective enzymes in the PA biosynthesis in heterologous yeast cell. Thus, this novel assay provides a powerful tool for quick identification and validation of a hit acyltransferase sequence, thereby contributing to delineating the precise map for the *de novo* glycerolipid biosynthesis in diverse crop species.

## Materials and Methods

### Yeast Growth Conditions

Yeast cells were cultured at 30°C in the synthetic minimal medium comprising 0.67% yeast nitrogen base, 2% glucose or galactose, and auxotrophic amino acids as needed for growth and as required for plasmid maintenance (Ausubel et al., 1994; Zheng and Zou, 2001). The genotypes of yeast strains frequently used in this study is shown in Supplementary Table S1.

### Construction of *GAT1* and *GAT2* Expression Vectors

The coding regions of *GAT1* (*YKR067w*) and *GAT2* (*YBL011w*) genes were PCR amplified with genomic DNA isolated from yeast BY4742 strain. BamH I and yeast Kozak sequence were included in forward primers (FP), and Xho I in backward primers (RP). FP and RP for *GAT1* amplification were 5′-GGATCCAACATGTCTGCTCCCGCTGCCGATCAT-3′ and 5′-CTCGAGTCATTCTTTCTTTTCGTGTTCTCT-3′; and those for GAT2, 5′-GGA TCCAACATGCCTGCACCAAAACTCACGGAG-3′ and 5′-CT CGAGCTACGCATC TCCTTCTTTCCCTTC-3′. The amplified DNA fragments were cloned into the pCR2.1 vector using TA cloning kit (Invitrogen). The resultant plasmids containing *GAT1* and *GAT2* were designated pCR2.1-GAT1 and pCR2.1-GAT2, respectively. To construct yeast expression vectors, the *GAT1* and *GAT2* genes were then excised from these two plasmids through digestion with BamH I and Xho I and inserted into the pYES2 vector (Invitrogen) at the respective restriction enzyme sites. The integrity of the resulting recombinant plasmids pYES2-GAT1 and pYES2-GAT2 were verified by sequencing.

### Construction of Yeast *gat1*Δ*gat2*Δ Double Mutant Bearing Episomal *GAT1* Gene

To construct the *gat1*Δ*gat2*Δ double mutant, a single *gat1*Δ mutant derived from BY4742 strain was chosen as a starting strain. The recombinant pYES2-GAT1 plasmid harboring *URA3* as a selection marker and *GAT1* gene under the control of *GAL1* promoter (*pGAL1::GAT1 URA3*) was transformed into *gat1*Δ to yield a new strain (*gat1*Δ+[*pGAL1::GAT1 URA3*]). *GAT2* gene in the resultant strain was then knocked out through replacing a 1605-bp fragment in the *GAT2* coding region with the amino acid selection marker gene *HIS3* to generate the double mutant *gat1*Δ*gat2*Δ+[*pGAL1::GAT1 URA3*], designated NIU8. It is noted that the *GAT2* knockout cassette transformed into the *gat1*Δ+[*pGAL1::GAT1 URA3*] strain comprises a *HIS3* selection marker that is flanked by two partial *GAT2* sequences, essentially as described previously (Zheng and Zou, 2001). Disruption of the *GAT2* gene was confirmed by PCR and sequencing. The *gat1*Δ+*pYES2* strain was used as a negative control.

### Construction of Recombinant YEplac181 Plasmids

To clone *GAT1* into YEplac181 plasmid that contains a *LEU2* selection marker, the entire transcription unit consisting of *GAT1* coding sequence, *GAL1* promoter and *CYC1* terminator was PCR amplified from the pYES2-GAT1 plasmid with a pair of primers containing Pst I and Sma I sites at the forward and backward primers, respectively. The resultant fragment was inserted between the two respective sites of YEplac181, generating YEplac181-GAT1. To construct recombinant YEplac181 plasmids harboring other genes, the coding sequence of each gene was substituted for that of *GAT1* between BamHI and XhoI sites in the above YEplac181-GAT1 vector. For those genes carrying internal BamH I or Xho I site, isocaudomers of these two enzymes were used to generate identical termini for cloning. The primers used are shown in Supplementary Table S2.

### *In Vivo* Complementation of NIU8

NIU8 harboring pYES2-GAT1 plasmid is a *URA3*^+^ strain. To substitute individual *AtGPAT* gene for the episomal *GAT1* gene, NIU8 was transformed with a recombinant YEplac181-AtGPAT plasmid (*pGAL1::AtGPAT LEU2*) using a lithium acetate-based standard protocol (Ausubel et al., 1994; Zheng and Zou, 2001). The resultant strain contained both pYES2-GAT1 and YEplac181-AtGPAT plasmids, thereby being *URA3*^+^ and *LEU2*^+^. It was then subjected to 5-fluoroorotic acid (5-FOA) counter-selection. URA3 allows for both positive and negative selection as it can complement uracil auxotrophy of the URA3-defective cells and meanwhile function in the conversion of the non-toxic 5-FOA compound to toxic 5-flurouracil (Boeke et al., 1984). The negative selection process was composed of two steps as described previously (Brown and Szostak, 1983): (i) culturing the cells from individual colony bearing pYES2-GAT1 and YEplac181-AtGPAT plasmids overnight (or until cell density reached an OD_600_ of about 5) in non-selective synthetic minimal medium supplemented with uracil (0.1 g L^-1^) and all essential amino acids to promote the loss of pYES2-GAT1 plasmid in a small percentage of the cells; and (ii) plating a portion of the resultant mixed cell population (10^6^–10^7^) onto yeast synthetic medium supplemented with agar (20 g L^-1^), galactose (20 g L^-1^), uracil (0.1 g L^-1^) and 5-FOA (1 g L^-1^) to kill those cells still containing pYES2-GAT1. The resulting colonies that were supposed to be *URA3*^-^ and *LEU2*^+^, were tested for the presence of heterologous *AtGPAT* gene and the absence of *GAT1* using PCR.

### Construction of a New pYES2 Plasmid Carrying *ADH1* Promoter

To test candidate genes for their capacity to rescue the ZAFU1 growth defect on glucose, a truncated 410-bp yeast alcohol dehydrogenase 1 promoter (*ADH1*) able to drive constitutive gene expression on various carbon sources (Tornow and Santangelo, 1990; Ruohonen et al., 1995) was used to replace the *GAL1* promoter in pYES2, which is a 2 μm circle plasmid of *S. cerevisiae* maintained at high copy numbers (Futcher, 1988). In addition, a kanamycin selection marker *Kan(R)* was inserted at the Nhe I site of the above modified pYES2 vector, which was designed for easy plasmid rescue, to yield a new shuttle vector, designated pYES2-Kan-ADH1. The integrity of the *ADH1* and *Kan* sequences was verified by sequencing. To perform ZAFU1-based complementation assays, individual genes were cloned into this vector. The primers used are shown in Supplementary Table S3.

### *In Vivo* Complementation of ZAFU1

Complementation of ZAFU1 with individual target gene was performed according to the following procedure. First, a target gene with a yeast Kozak sequence [(G/A)NNATGG] included for good translation initiation was cloned into the multiple cloning site of the pYES2-Kan-ADH1 vector, which comprises Hind III, Sac I, BamH I, EcoR I, Xho I, and Xba I. Second, the ZAFU1 strain was grown at 30°C in yeast synthetic medium containing galactose (20 g L^-1^) and uracil (0.1 g L^-1^) in the absence of histidine and leucine until the cell density reached an OD_600_ of approximately 1.0 ∼ 1.2. Subsequent transformation of ZAFU1 with the recombinant pYES2-Kan-ADH1 plasmid was carried out using a lithium acetate-based standard protocol (Ausubel et al., 1994; Zheng and Zou, 2001), with empty vector as control. Last, the transformed cells were plated onto yeast synthetic medium containing agar (20 g L^-1^) and either glucose (20 g L^-1^) or galactose (20 g L^-1^) as carbon source in the absence of uracil, histidine, and leucine, and incubated at 30°C for a period of time (usually 2 ∼ 5 days). The resultant colonies were restreaked on the same medium to obtain pure cultures, and then used in other experiments.

## Results

### Characteristics of the *gat1*Δ*gat2*Δ Double Mutant Bearing the Episomal *GAT1* Gene

**FIGURE 1:**
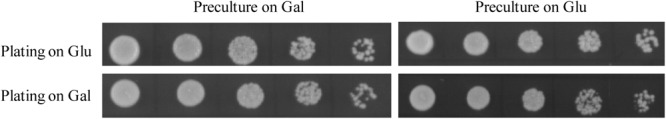
Growth behavior of NIU8 (*gat1*Δ*gat2*Δ+[*GAL1:GAT1 URA3*]) on SC medium containing glucose (Glu) or galactose (Gal). The NIU8 cells were precultured overnight in liquid SC medium containing Glu or Gal in the absence of histidine and uracil, and then serial diluted (1:5) with the initial optical density (at 600 nm, OD_600_) of 0.5 and plated onto SC medium containing Glu or Gal in the absence of histidine and uracil for 2 days. Essentially identical results were obtained in three independent experiments.

One challenge of *in vivo* complementation assay is to design a mutant strain whose growth defect is rescued only by expression of a sequence of interest encoding genuine GPAT. Based on the previous findings that disruption of both *GAT1* and *GAT2* genes coding for GPAT enzymes led to lethality in *S. cerevisiae* (Zheng and Zou, 2001; Zaremberg and McMaster, 2002), we have developed a conditional lethal *gat1*Δ*gat2*Δ double mutant (*gat1*Δ*gat2*Δ+[*pGAL1::GAT1 URA3*]) on the background of BY4742 strain. Intriguingly, NIU8 could grow not only on galactose but also on glucose despite that the *GAL1* promoter used for driving the episomal *GAT1* gene expression was normally repressed by glucose (**Figure 1**). This is in a sharp contrast to the previous report that the conditional lethal double mutant CMY228 on the genetic background of W303 did not seem to grow on glucose despite that the same *GAL1* promoter and episomal *GAT1* gene were present in CMY228 (Zaremberg and McMaster, 2002). One explanation could be that the disparity in their growth behaviors is due to the difference in the genetic background between BY4742 and W303 (Ralser et al., 2012). However, we cannot rule out another possibility that the copy number of the episomal plasmid in NIU8 might be higher than that of CMY228, leading to potentially high levels of leaky expression of the episomal *GAT1* gene on glucose. This view is supported by our RNA-Seq experiment revealing the presence of *GAT1* transcripts in the NIU8 cells growing on glucose, albeit at a 20-fold lower level compared to that on galactose. In light of the previous finding that deficiency of Gat1p or Gat2p in yeast cells can activate a compensation mechanism to boost GPAT activity through post-translation modification (Bratschi et al., 2009), it is plausible to assume that leaky expression of the episomal *GAT1* gene on glucose may generate sufficient GPAT activity to sustain the NIU8 cell growth on glucose.

**FIGURE 2:**
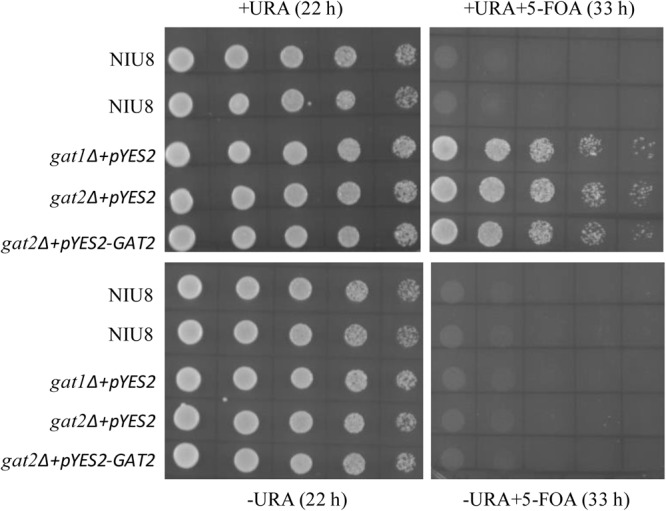
Comparison of growth behaviors between the *gat1*Δ*gat2*Δ double mutant NIU8 and the single mutants upon 5-FOA treatment. Cell cultures of different strains were incubated with non-selective liquid medium containing uracil (URA) overnight and then serial diluted (1:4) with the initial optical density (at 600 nm, OD_600_) of 0.5 and plated onto Gal-containing medium with (+) or without (-) URA or 5-FOA for 22 or 33 h. Essentially identical results were obtained in each of three independent experiments.

To test if NIU8 is fully reliant on the *GAT1* gene in the episomal pYES2 plasmid (*pGAL1::GAT1 URA3*) for its survival and growth, we carried out 5-fluoroorotic acid (5-FOA) counter-selection of this strain (Brown and Szostak, 1983; Boeke et al., 1984) as described in Section “Materials and Methods.” In the absence of 5-FOA, cell populations of various genotypes including NIU8, *gat1*Δ harboring pYES2, and *gat2*Δ harboring either pYES2 or the episome of *pGAL1::GAT1 URA3* could all proliferate well with or without uracil, whereas they could not live on 5-FOA in the absence of uracil. However, while cell cultures of those single mutants could thrive in the simultaneous presence of 5-FOA and uracil, growth cessation occurred for the double mutant NIU8 under this condition (**Figure 2**). The above results clearly rule out any possible insertions of a functional *GAT1* sequence into yeast genome to sustain the growth of the *gat1*Δ*gat2*Δ double mutant. Rather, our data indicate that the *URA3* and *GAT1* genes are kept intact in the *pGAL1::GAT1 URA3* episome and that the episomal *GAT1* gene is indispensable for sustaining NIU8 growth, corroborating our previous finding that simultaneous inactivation of *GAT1* and *GAT2* is lethal to yeast (Zheng and Zou, 2001).

### A False-Positive Problem Associated With the NIU8-Based Complementation Assay

Since NIU8 can grow on both galactose and glucose, complementation of this strain does not allow for direct phenotypic screen of GPAT genes. But the fact that the episomal *GAT1* gene is essential for sustaining NIU8 growth suggests that if its replacement with a gene of interest can rescue the growth defect of the *gat1*Δ*gat2*Δ double mutant, the enzyme encoded by this gene may mimic a certain aspect of Gat1p in the initial step of PA synthesis. Thus, when combined with 5-FOA counter-selection to purge the preexisting *GAT1* episome, the NIU8-based complementation could still be used for functional dissection of GPAT genes. However, in the following experiments with NIU8 carrying individual target genes, a false-positive screen was encountered.

5-FOA counter-selection of NIU8 transformants bearing the control plasmid YEplac181 did not yield a single colony in several repeated experiments. In contrast, the assays with yeast *PST1/CST26/YBR042c* coding for *sn*-2-acyl-1-lysophosphatidylinositol acyltransferase (Le Guédard et al., 2009), *Arabidopsis LPAT4* for a putative LPAT (Kim et al., 2005) and *At5g13760* for a putative choline transporter (Zhou and Gitschier, 1997) yielded approximately 260, 150, and 100 colonies, respectively, from an initial inoculum of 5.0 × 10^7^ cells on the glucose-containing SC medium supplemented with uracil and 5-FOA. Surprisingly, however, PCR analysis on randomly selected colonies revealed that in addition to individual target gene, the intact *GAT1* coding sequence with the expected size was amplified in all the colonies (**Table 1**). One explanation is that a very low copy number of the *GAL1::GAT1 URA3* plasmid is still present in some of these colonies (**Table 1**) so as to yield sufficiently high GPAT activity but fairly low conversion of 5-FOA to the toxic product by URA3. Alternatively, the *GAT1* coding sequence from the plasmid might be integrated into yeast genome in some other colonies during 5-FOA counter-selection. Thus, the use of yeast native *GAT1* gene as a component of the episome to maintain cell growth and proliferation of the *gat1*Δ*gat2*Δ double mutant seemed to pose a problem of false-positive characterization of target sequences. This problem renders the phenotypic screen ineffective and therefore necessitates the development of an alternative screen system.

**Table 1:** False positive identifications of target genes associated with the NIU8-based complementation assay.

Target gene	Organism	Peptide size	Function	Colony number	PCR product		
					*URA3*	*GAT1*	Target gene
Control (YEplac181)				0	NA	NA	NA
*Atlg75020*	*A. thaliana*	378 aa	LPAT4	153	3/3^∗^	3/3	3/3
*YBR042c*	*S. cerevisiae*	397 aa	Pst1p, Cst26p	269	0/3	15/15	15/15
*At5g13760*	*A. thaliana*	569 aa	Putative choline transporter	105	1/3	15/15	15/15

*Approximately 5 × 10^*7*^ cells were plated onto minimal medium containing uracil and 5-FOA but lacking leucine and histidine. PCR analysis showed that all of the randomly chosen colonies contained *GAT1* gene that is likely to be originated from the preexisting episome [*GAL1::GAT1 URA3*] in NIU8. ^∗^The numerator denotes the total number of colonies positive for the tested gene and the denominator for the total number of colonies tested.*

### Generation of a Novel Conditional Lethal *gat1*Δ*gat2*Δ Mutant Bearing Heterologous *AtGPAT1* Gene

One underlying mechanism behind the false-positive problem associated with the NIU8-based complementation could be ascribed to intrinsic properties of Gat1p enzyme in the native cellular environment that permit yeast cells to boost the catalytic activity under the circumstance where *GAT1* is expressed at low levels (Bratschi et al., 2009). Frequently, the catalytic activity of a GPAT enzyme is most highly expressed in its native cell type, whereas it is lower in a heterologous cell system. Thus, we proposed that one strategy for resolving the above problem is to curb the GPAT activities in the *gat1*Δ*gat2*Δ double mutants through replacement of yeast *GAT1* episome with a novel episome bearing a heterologous GPAT gene. We then investigated the feasibility of this strategy, referred to as the Eradication of Excess Capacity strategy, using *Arabidopsis AtGPAT1* gene. This is based on our previous finding from *in vitro* GPAT assay showing that AtGPAT1, when heterologously expressed in yeast *gat1*Δ cell, had 10-fold lower GPAT activities than yeast Gat1p and Gat2p (Zheng and Zou, 2001; Zheng et al., 2003).

**FIGURE 3:**
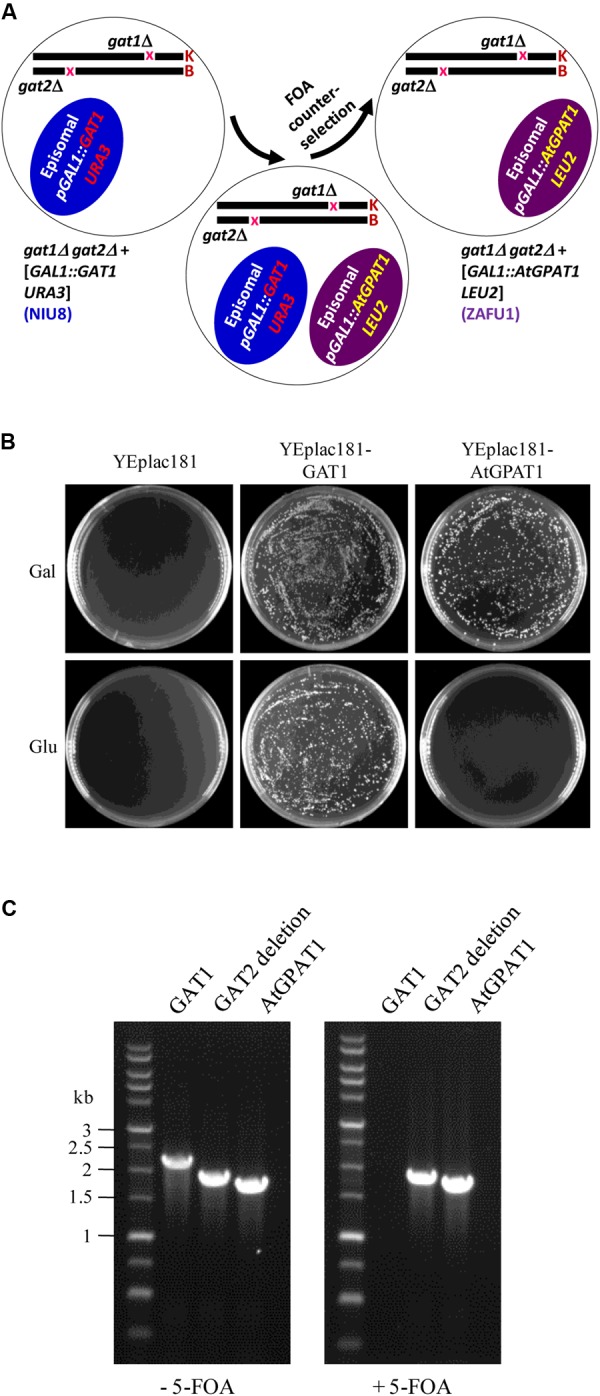
Construction of the *gat1*Δ*gat2*Δ double mutant harboring an episomal *AtGPAT1* gene. **(A)** A schematic diagram of the complementation procedure involving 5-FOA counter-selection. K and B: yeast chromosomes. **(B)** Complementation of NIU8 with the YEplac181 plasmid carrying *AtGPAT1* or *GAT1* gene as a control under the control of *GAL1* promoter. YEplac181 has a *LEU* selection marker. The transformants were grown on the medium spiked with uracil, 5-FOA and Gal (or Glu) but lacking histidine and leucine. Essentially identical results were obtained across independent experiments. **(C)** PCR analysis of the strain resulting from the transformation of NIU8 with YEplac181-AtGPAT1 plasmid before (-) and after (+) 5-FOA counter-selection for the presence of *AtGPAT1* and the episomal *GAT1* gene. It is noted that *GAT1* is from the episome *GAL1:GAT1 URA3* but not from *GAT1::kanR* in the NIU8 genome. The defective *GAT2* gene into which the *HIS3* selection marker is inserted was used as internal control, with its size of 1840 bp vs. 2280 bp for the wild type.

To substitute *AtGPAT1* for the episomal *GAT1* gene, the recombinant YEplac181 plasmid (*pGAL1:AtGPAT1 LEU2*) carrying the *LEU2* selection marker and *AtGPAT1* gene under the control of *GAL1* promoter was transformed into the NIU8 strain. The resulting strain harboring two different plasmids underwent the non-selection culture and 5-FOA counter-selection to discard the recombinant pYES2 plasmid (*pGAL1:GAT1 URA3*) (**Figure 3A**). In the presence of galactose, thousands of colonies were formed on the SC medium containing 5-FOA and uracil. In sharp contrast, no colonies appeared when glucose was supplemented to repress *AtGPAT1* expression (**Figure 3B**). PCR analysis on randomly selected colonies showed that all of them lack *GAT1*, but bear *AtGPAT1* gene (**Figure 3C**). This result suggests that the final resultant strain has lost the *pGAL1:GAT1 URA3* episome and instead retained *pGAL1:AtGPAT1 LEU2* and that *AtGPAT1* has the capacity to take the place of the episomal *GAT1* to rescue the *gat1*Δ*gat2*Δ growth defect. Hence, this strain has the genotype of *gat1*Δ*gat2*Δ+[*pGAL1::AtGPAT1 LEU2*], designated ZAFU1.

Strikingly, when the same NIU8 strain transformed with the recombinant YEplac181 plasmid carrying yeast *GAT1* gene (*pGAL1:GAT1 LEU2*) underwent the same selection process as that with *pGAL1:AtGPAT1 LEU2*, thousands of colonies could be formed on both galactose and glucose (**Figure 3B**). Our data strongly suggest that leaky expression of the episomal yeast *GAT1* gene under conditions of glucose repression can yield sufficient GPAT activities to sustain the *gat1*Δ*gat2*Δ mutant growth, thus highlighting the necessity of using the Eradication of Excess Capacity strategy to develop a genetic complementation assay.

As expected, ZAFU1 was characterized by slow growth on galactose-containing synthetic minimal medium. Its doubling time during the logarithmic phase of growth was approximately 4 h, which is in a sharp contrast to about 2 h for its isogenic wild type and two single *gat1*Δ and *gat2*Δ mutants. This characteristic feature may be explained by taking into account that only high levels of transcriptional expression of *AtGPAT1* on galactose could generate sufficiently high GPAT activities to sustain the double mutant growth, whereas low levels of its expression on glucose failed to do so. Thus, it is very likely that this novel mutant is less prone to growing non-specifically under glucose-repressing conditions.

### Characteristics of a Novel ZAFU1-Based Complementation Assay

To develop a ZAFU1-based complementation system for functional analysis of GPAT sequences, we constructed a new shuttle vector, designated pYES2-Kan-ADH1. It features the kanamycin selection marker (Kan) useful for plasmid rescue and yeast alcohol dehydrogenase 1 promoter (*ADH1*) to drive constitutive gene expression on glucose (Tornow and Santangelo, 1990; Ruohonen et al., 1995). This new assay does not involve 5-FOA counter-selection.

**FIGURE 4:**
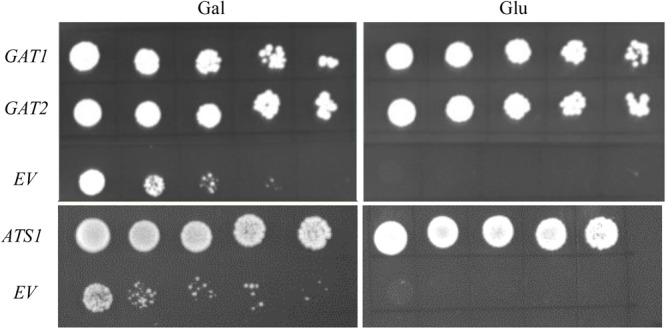
Complementation of the conditional lethal mutant ZAFU1 (*gat1*Δ*gat2*Δ+[*pGAL1::AtGPAT1 LEU2*]) with known genes encoding GPAT enzyme. Various cultures of ZAFU1 cells carrying pYES2-Kan-ADH1 (*EV*) or the recombinant plasmids with *GAT1, GAT2*, and *ATS1*, respectively, were serial diluted (1:4) with the initial optical density (at 600 nm, OD_600_) of 0.5 and plated onto Gal- or Glu-containing medium in the absence of histidine, leucine and uracil for 3 days. Essentially identical results were obtained in three independent experiments.

Its validity was initially assessed by testing nine genes with well-defined metabolic functions in phospholipid biosynthesis for their capacity to rescue the PA synthetic defect in ZAFU1 on glucose. The respective protein products can be divided into four distinct categories: (i) GPAT (*ATS1, GAT1*, and *GAT2*) (Nishida et al., 1993; Zheng and Zou, 2001); (ii) LPAT (*SLC1, LPAT4*/*At1g75020*) (Nagiec et al., 1993; Kim et al., 2005); (iii) *sn-2*-acyl-1-lysophosphatidylinositol acyltransferase (*PST1/CST26/YBR042c*) (Le Guédard et al., 2009); and (iv) choline transporter-like protein (*At5g13760, At1g25500*, and *YOR161c*) (Zhou and Gitschier, 1997). Each gene was driven under the control of *ADH1* promoter and transformed into ZAFU1. Complementation with either yeast *GAT1* or *GAT2* gene yielded more than five thousands of colonies on glucose, and cell cultures of these colonies at various densities were grown well on glucose (**Figure 4**). Similar effect was exerted by an *Arabidopsis ATS1* sequence coding for the mature ATS1 protein devoid of a 90 aa-long transit peptide at the N-terminal region (Nishida et al., 1993) (**Figure 4**). Noticeably, ATS1 is a plastidial soluble GPAT that is structurally unrelated or distantly related to membrane-bound GPAT and does not adhere to cellular membranes (Murata and Tasaka, 1997). Thus, the capacity of ATS1 to restore the ZAFU1 growth defect is ascribed to its catalytic activity, as opposed to its physical interaction with yeast membrane-bound enzymes in the PA biosynthetic pathway. In a sharp contrast, genes of other three categories did not yield single colonies, which include yeast *PST1* and *Arabidopsis LPAT4* and *At5g13760* that were previously tested false-positive in the NIU8-based complementation assay. Collectively, our data indicate that this novel assay is capable of distinguishing GPAT-encoding genes from those coding for other proteins, thereby demonstrating its high specificity for GPAT genes and in turn highlighting its validity and utility in functional characterization of GPAT sequences.

**FIGURE 5:**
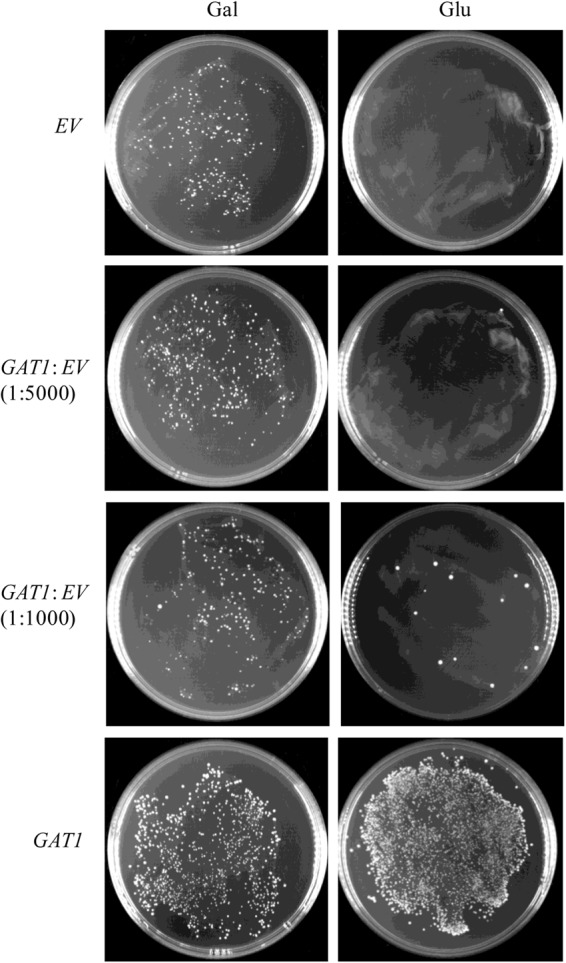
Sensitivity of a ZAFU1-based complementation Assay for functional characterization of *GAT1*. ZAFU1 cells were transformed with pYES2-Kan-ADH1 (*EV*) or the recombinant plasmid bearing *GAT1* (*GAT1*), or a mixed population of the plasmids. One-sixth and five-sixths of the whole transformants were plated onto Gal- or Glu-containing medium, respectively. Similar results were obtained in three independent experiments.

To assess its sensitivity, the recombinant pYES2-Kan-ADH1 plasmid bearing *GAT1* gene was mixed with the respective empty vector in ratios of 1:1000 and 1:5000 and then transformed into ZAFU1 to test the ability of the system to recover the *GAT1*-containing plasmid. As shown in **Figure 5**, the formation of colonies was readily observed as the result of transformation with a mixed population of the two plasmids (1:1000). Moreover, the recovered plasmids from all of three randomly selected colonies did confer kanamycin resistance in the transformed DH_5α_ bacterial cells. These results suggested that the novel assay has an acceptable sensitivity for accurate screening of GPAT genes.

### Properties of Heterologously Expressed *Arabidopsis* AtGPAT Family Members

To demonstrate the utility of this novel complementation assay, we tested individual members of *Arabidopsis* AtGPAT family for the capacity to mimic yeast Gat1p with respect to the restoration of the PA biosynthetic defect in the *gat1*Δ*gat2*Δ double mutant. This family is comprised of eight members (Zheng et al., 2003; Chen et al., 2011; Yang et al., 2012). Thus far, information regarding their *in vivo* acylation properties is lacking although five members of this family were shown to predominantly catalyze *in vitro* acylations on glycerol-3-phosphate at the *sn*-2 position (Yang et al., 2012). In addition, the other three members AtGPAT2/3/7 (for simplicity, placing them together and separating by slash when three or more members appear consecutively) have not yet been successfully assayed *in vitro* (Yang et al., 2012).

**FIGURE 6:**
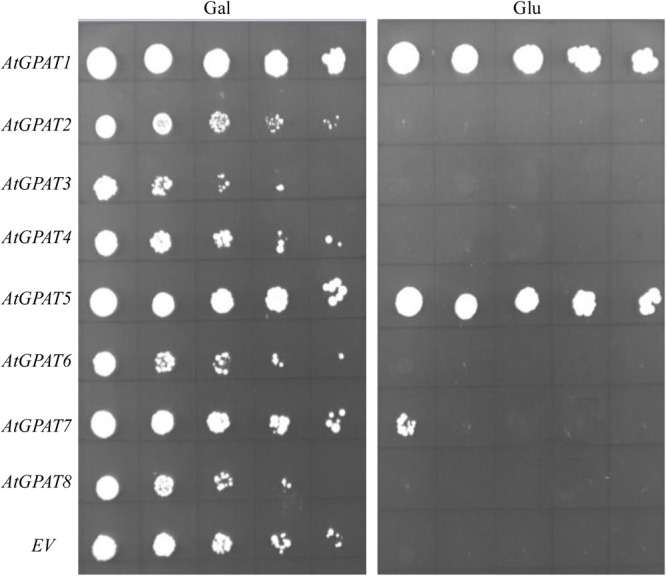
Complementation of the conditional lethal mutant ZAFU1 (*gat1*Δ*gat2*Δ+[*pGAL1::AtGPAT1 LEU2*]) with individual *AtGPAT* genes. ZAFU1 was transformed with individual genes under the control of *ADH1* promoter. The resultant colonies grown on Gal were streaked to obtain pure cell isolates. Cell cultures from individual colonies were serial diluted (1:5) and plated on synthetic medium lacking three amino acids (uracil, histidine, and leucine) in the presence of Gal or Glu. Repeated experiments produced the same results. *EV*, empty vector (pYES2-Kan-ADH1) as control.

Complementation of ZAFU1 with individual *AtGPAT* gene under the control of *ADH1* promoter was conducted as described above. To provide a better comparison of the effects of individual genes, a limiting dilution assay on cell cultures of various strains initially maintained on galactose was utilized. It was evident that expression of either *AtGPAT1* or *AtGPAT5* on glucose could allow ZAFU1 to grow at a rate similar to that of yeast *GAT1* or *GAT2* (**Figure 6**), suggesting that these two *Arabidopsis* genes could generate sufficient GPAT activities for sustaining rapid cell growth. GPAT7 was also shown to possess a significant albeit low activity for the restoration of the lethal phenotype (**Figure 6**). However, the other five genes *AtGPAT2/3/4/6/8* failed to complement the ZAFU1 growth defect on glucose (**Figure 6**).

**FIGURE 7:**
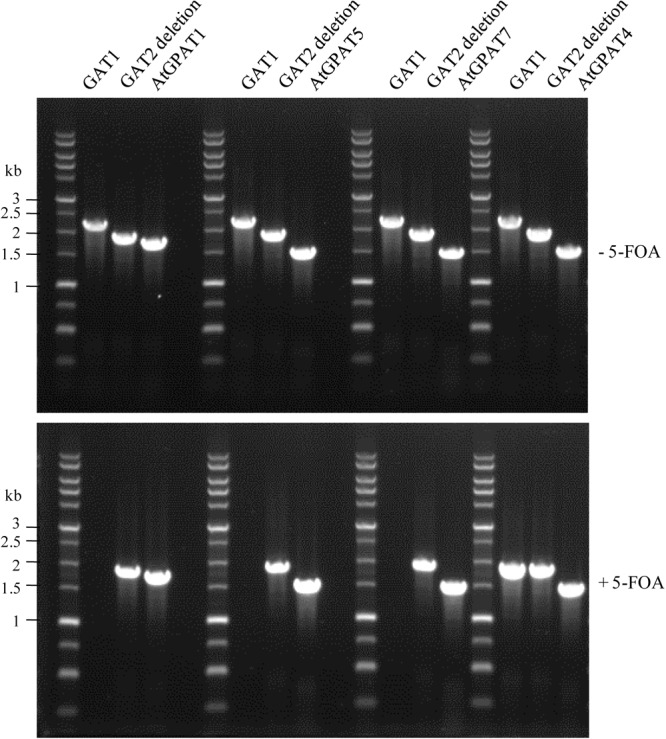
PCR verification of replacement of the episomal *GAT1* gene with individual *AtGPAT* gene in the NIU8 mutant (*gat1*Δ*gat2*Δ+[*pGAL1::GAT1 URA3*]). Complementation of NIU8 with the YEplac181 plasmid carrying individual *AtGPAT* gene under the control of *GAL1* promoter (*pGAL1::AtGPAT LEU2*) was conducted, and the transformants were PCR analyzed for the presence of yeast episomal *GAT1* and individual *AtGPAT* gene before (-) and after (+) 5-FOA counter-selection, essentially as described in **Figure 3**. *AtGPAT1* shown in **Figure 3** was used as a positive control. The defective yeast *GAT2* gene into which the *HIS3* selection marker is inserted was used as internal control, with its size of 1840 bp vs. 2280 bp for the wild type *GAT2*. Essentially identical results were obtained across independent experiments.

Noticeably, the functionality of *AtGPAT1/5/7* genes manifested in the ZAFU1-based assay could be recapitulated in the NIU8-based assay. YEplac181 plasmids harboring individual *AtGPAT* genes under the control of *GAL1* promoter (*pGAL1:AtGPAT LEU2*) were transformed into NIU8, followed by 5-FOA counter-selection to purge the preexisting *GAT1* episome, as described previously. It was evident that while no colonies appeared under the condition of glucose repression, replacement of the episomal *GAT1* gene in NIU8 with *AtGPAT5* could yield more than five thousands of colonies on galactose, which is very similar to that of *AtGPAT1* (**Figure 3B**). As for *AtGPAT7*, the number of colonies formed on galactose could reach approximately one-fiftieth that of *AtGPAT5*, and these colonies appeared to grow at a slower rate than those for *AtGPAT5*. PCR analysis revealed the absence of the episomal *GAT1* gene but the presence of one of the three genes *AtGPAT1/5/7* in the resulting colonies (**Figure 7**). Support for the loss of *pGAL1:GAT1 URA3* episome came from the observation that the resultant colonies could not grow without uracil. These results indicate that *AtGPAT1/5/7* have the capacity to rescue the PA biosynthetic defect upon their substitution for the episomal *GAT1* gene in NIU8. In contrast, such capacity appeared to be lacking for the five other genes *AtGPAT2/3/4/6/8* since the complementation assays for these genes only occasionally yielded a few colonies and all of them were PCR identified as *GAT1*-positive for some unknown reasons and deemed false positives. As an example, a false-positive screen of *AtGPAT4* was shown in **Figure 7**. Thus, the *in vivo* roles of these genes could not be readily determined.

Overall, each *AtGPAT* gene appeared to exert reproducible effects on restoration of the growth defect of the *gat1*Δ*gat2*Δ double mutant in the two distinct *in vivo* assays, thus underscoring the validity of our data. It is therefore concluded that AtGPAT1/5/7 possess the capacity for PA biosynthesis in heterologous yeast cells, albeit at varying degrees. Nevertheless, the ratio of *sn*-2 to *sn*-1 lysophosphatidic acid (LPA) formed by these enzyme in yeast cells remains unknown.

## Discussion

Owing to the existence of a certain degree of conservation of glycerolipid biosynthesis between unicellular and multicellular eukaryotes, functional complementation of yeast mutants has been successfully employed to identify many heterologous genes involved in this primary metabolism. However, the main criticism applied to yeast *in vivo* assay is the possibility of a high number of false positive identifications. The exact rate of false positive results is not known, but earlier estimates were as high as 90% (Zhou and Gitschier, 1997). In this study, we developed a novel and convenient yeast complementation assay with high specificity for functional analysis of GPAT sequences.

### Rational Design and Utility of a Novel Yeast Complementation System for Functional Dissection of GPAT Genes

One key to developing an effective *in vivo* assay for GPAT is to solve the difficulty in generating a mutant strain whose phenotypic defect is specifically caused by GPAT deficiency and rescued only in the presence of target sequences having genuine GPAT activities. The lethality caused by simultaneous inactivation of *GAT1* and *GAT2* genes allows for the design of the conditional lethal *gat1*Δ*gat2*Δ mutants whose survival and growth is reliant on the expression of an episomal GPAT-encoding gene. Theoretically, loss of the episome or complete suppression of the episomal GPAT expression is supposed to render the cell unviable due to complete deficiency of the *sn-1* acyltransferase activities mediating the first step of glycerolipid biosynthesis. On the other hand, a GPAT-encoding sequence, when expressed in the mutant or upon its substitution for the existing episome, is expected to complement the growth defect. Yet this seemingly simple process could be complicated due to some unforeseen circumstances.

Our RNA-Seq study on the conditional lethal *gat1*Δ*gat2*Δ double mutant NIU8 showed that there existed leaky expression of *GAL1* promoter-driven episomal *GAT1* gene under glucose-repressing conditions. This factor is very likely to account for the ability of NIU8 to grow on glucose, whereas it is unlikely that an unknown mutation, if any, introduced into NIU8 in the course of the strain construction constitutes a mechanism of NIU8 growing on glucose. The reasons are twofold. First, NIU8 could not survive without the episome *pGAL1:GAT1 URA3* (**Figure 2**). Second, its replacement with that of *pGAL1:AtGPAT1 LEU2* carrying *Arabidopsis AtGPAT1* under the control of the same *GAL1* promoter abolished the capacity of the double mutant to grow on glucose while allowing the mutant to form colonies on galactose (**Figure 3B**). In sharp contrast, when it was replaced by the episome *pGAL1:GAT1 LEU2*, the double mutant could grow under conditions of glucose repression (**Figure 3B**). Thus, we postulate that one underlying mechanism restoring the NIU8 growth under glucose-repressing conditions involves intrinsic capacities of the *GAT1* gene in the native cellular environment to facilitate expression of sufficiently high GPAT activity for yeast growth even at low levels of its transcript accumulation. This view is supported by the previous finding demonstrating that deficiency of either Gat1p or Gat2p in yeast can activate a compensation mechanism to boost GPAT activity through post-translation modification of these two enzymes (Bratschi et al., 2009). In light of this view, one could envision that under certain circumstances where leaky expression of episomal *GAT1* gene or its integration into yeast genome occurs due to certain harsh selection pressure (such as 5-FOA counter-selection), a problem of false-positive screen of target genes would be encountered with some *gat1*Δ*gat2*Δ double mutants like NIU8 strain and particularly with those carrying high copy number of the episomal *GAT1* plasmids. Such scenario did occur in our studies with three lipid-related genes (yeast *PST1, Arabidopsis LPAT4* and *At5g13760*) since besides individual target gene, the intact episomal *GAT1* coding sequence was found to be present in all of the randomly selected colonies resulting from the complementation of NIU8 with these genes (**Table 1**). This phenomenon poses dire challenges for determining whether a candidate sequence has GPAT activity, thereby rendering the phenotypic screen ineffective.

The present study demonstrated that the false positive problem could be circumvented using the Eradication of Excess Capacity strategy mentioned previously. It involves the replacement of the native episomal *GAT1* gene in NIU8 with heterologous *AtGPAT1* gene to curtail GPAT activities in the *gat1*Δ*gat2*Δ double mutant. The resultant ZAFU1 strain is unable to grow under the condition of glucose repression. One mechanism underlying this phenomenon could be ascribed to the low catalytic activity of AtGPAT1 heterologously expressed in yeast (Zheng et al., 2003), suggesting that its leaky expression in yeast cell on glucose, if any, is less prone to conferring sufficient GPAT activities for the mutant growth. Thus, when the ZAFU1 strain was coupled with our newly designed shuttle vector permitting the expression of target genes on glucose, we could develop a novel complementation assay that is suitable for one-step phenotypic screening of GPAT-encoding genes on glucose without 5-FOA counter-selection. Its high specificity and robustness for functional dissection of GPAT sequences were illustrated by the following lines of evidence. First, in the absence of any tested genes, the ZAFU1 mutant transformed with the empty vector is unable to grow on glucose under laboratory conditions (**Figure 4**). Second, expression of those GPAT-encoding genes (yeast *GAT1* and *GAT2* and *Arabidopsis ATS1*) under the control of *ADH1* promoter could readily rescue the ZAFU1 growth defect on glucose (**Figure 4**). Third, neither the genes encoding other acyltransferases than GPATs (for instance, yeast *SLC* and *PST1*) nor other lipid-related genes (yeast *YOR161c* and two *Arabidopsis* choline transporter-like sequences, *At5g13760* and *At1g25500*) could restore the defect. While complementation of ZAFU1 with these genes did not yield a single false-positive colony on glucose, false-positive screens for three of these genes have been shown to occur in the NIU8-based assay (**Table 1**). Together, our data demonstrate that the novel ZAFU1-based assay is capable of effectively distinguishing GPAT-encoding genes from those of other fatty acyltransferases and lipid-related sequences, thereby highlighting its high specificity for GPAT enzyme.

It is noted that high specificity of a complementation system may sometimes sacrifice its sensitivity. This seems to be the case for our novel assay as the transformation efficiency of the ZAFU1 strain is relatively low compared with that of NIU8. Nevertheless, the low sensitivity can often be compensated for by increased amounts of plasmids and competent cells used for transformation. As shown in **Figure 5**, our novel assay could readily distinguish one *GAT1*-containing plasmid from 1000 empty plasmids. Thus, the sensitivity of our assay does not seem to be a problem in routine applications.

In sum, the present Eradication of Excess Capacity strategy has led to a rational design of the ZAFU strain and the development of the ZAFU1-based *in vivo* assay that possesses high specificity for rapid analysis of GPAT genes and circumvents the false positive problem encountered with the complementation of the *gat1*Δ*gat2*Δ double mutant bearing the native *GAT1* gene. Its utility has been exemplified through analyzing *Arabidopsis AtGPAT* gene family, as will be further discussed in next section. We anticipate that the present strategy can be readily adaptable to the development of many other complementation assays for functional characterization of heterologous genes.

### Potential Functional Distinctness Among Different Members of *Arabidopsis AtGPAT* Gene Family in PA Synthesis in Yeast

Substantial evidence has implicated the pivotal roles of the *AtGPAT* family in lipid polyester synthesis (Beisson et al., 2007; Li et al., 2007; Mañas-Fernández et al., 2010; Yang et al., 2012; Petit et al., 2016). Five members of this family have been shown to prefer to catalyze *in vitro* acylation of glycerol-3-phosphate at the *sn*-2 position instead of *sn*-1. But the remaining three members AtGPAT2/3/7 have not yet been successfully assayed *in vitro* (Yang et al., 2012). To fully understand how different members of this family coordinately mediate the regulation of various lipid processes that share the common precursor LPA, we examined the *in vivo* properties of these enzymes in the heterologous system by taking full advantage of yeast complementation assays developed in this study.

The two distinct *in vivo* assays based on NIU8 and ZAFU1, respectively, revealed that there existed potential functional distinctness among different members of the *AtGPAT* gene family in the ability to restore the PA biosynthetic defect in the yeast mutants. When heterologously expressed under the control of galactose-inducible *GAL1* promoter, *AtGPAT1/5/7* could replace the episomal *GAT1* gene in the *gat1*Δ*gat2*Δ double mutant NIU8 to complement the growth defect. In contrast, such capacity was not registered with the other five members *AtGPAT2/3/4/6/8*. Likewise, when expressed under the control of the constitutive *ADH1* promoter, *AtGPAT1/5/7* could complement the PA biosynthetic defect in ZAFU1 on glucose, albeit at varying degrees, whereas this was not seen for *AtGPAT2/3/4/6/8*. Thus, our combined data appeared to readily distinguish *AtGPAT1/5/7* from other family members.

The failure of AtGPAT4/6/8 to complement the mutant growth defect seems to be in accord with their dual GPAT and phosphates activities that lead to conversion of glycerol-3-phosphate into LPA and immediately into monoacylglycerol (MAG) (Yang et al., 2010, 2012), thereby rendering a tiny amount of LPA for PA synthesis. At present, it is unclear whether yeast has sufficient MAG acyltransferase activity to convert MAG to diacylglycerol for phospholipid synthesis. Alternatively, such outcome associated with AtGPAT4/6/8 may be linked to their unique substrate specificities. For instance, while AtGPAT5 has broad substrate specificity, AtGPAT6 and AtGPAT8 prefer hydroxy-acyl-CoA or DCA-CoA over 16:0-, 18:0-, or 18:1-CoA (Yang et al., 2012). Considering that yeast cells contain only four major types of fatty acyl-CoA (16:0, 16:1, 18:0, and 18:1), these enzyme might inefficiently assemble them into glycerol-3-phosphate to yield LPA. Nevertheless, we cannot rule out other unknown mechanisms underlying their performance.

It should be pointed out that the manifested functionality of each enzyme can be difficult to distinguish from its role as *sn*-2 GPAT, which shows the preference for the *sn-2* acylation of glycerol-3-phosphate in the *in vitro* assays (Yang et al., 2010, 2012). One or more of the following mechanisms could be operated in yeast cells: (i) direct conversion of the 2-LPA isomer to PA; (ii) isomerization of 2-LPA to 1-LPA; and (iii) coexistence of *sn*-1 and *sn*-2 GPAT activities for these enzymes in yeast cells. Further studies will be required to clarify which explanation is germane to the actual *in vivo* situation.

It is also worth noting that despite the existence of a certain degree of conversation of the PA biosynthetic pathway between yeast and plants, the authentic roles of different members in PA synthesis in the native *Arabidopsis* species cannot be simply predicted based on their functions in yeast cells, but need to be experimentally clarified given that plant cells have more complex genetic interactions than yeast. It is therefore cautioned that the potential overlapping roles different members of the *AtGPAT* gene family play in PA synthesis in yeast cannot be used to muddle their distinctions, especially in the native cellular environment. To delineate functional redundancy and divergence of different variants of homoeologous, homologous, and paralogous acyltransferase sequences within the glycerol acyltransferase gene family in diverse allopolyploid crop species, it is essential to conduct *in planta* dissection of the effects of single, double and even triple or quadruple null mutations on various metabolic processes. Nevertheless, the present yeast complementation assay would help discern the functions of individual plant GPAT genes to a varying degree. In addition, our present study may open new clues for future studies aimed to delineate the interconnections between polyester and membrane glycerolipid biosynthetic pathways. It merits future efforts to investigate whether the pools of acylglycerol precursors for polyester synthesis are exchangeable with those for polar and neutral glycerolipids.

## Author Contributions

JL, YM, and YL completed most of the experiments. ZZ conceived the research plan, supervised the experiments, and participated in experimental design and data analysis. XH, LN, and YW participated in the construction of yeast double knockout mutants. HL and YG performed the screening of target genes. ZZ, JL, YM, and YL wrote this article with input from all the authors. All authors reviewed and approved this submission.

## Conflict of Interest Statement

The authors declare that the research was conducted in the absence of any commercial or financial relationships that could be construed as a potential conflict of interest.
