# Supplementary material for: A Novel Complementation Assay for Quick and Specific Screen of Genes Encoding Glycerol-3-Phosphate Acyltransferases
Source: Front Plant Sci. 2018 Mar 19;9:353. doi: 10.3389/fpls.2018.00353 (PMC5867339; doi:10.3389/fpls.2018.00353)
Supplement: Supplementary file 1 [file Table_1.PDF]

Table S1. The genotypes of yeast strains frequently used in the present study.

Strain	Genotype	Abbreviated form
BY4742	*MATα his3 leu2 met15 ura3*	
gat1 Δ	*MATα his3 leu2 met15 ura3 YKR067w::kanMX4*	
gat2 Δ	*MATα his3 leu2 met15 ura3 YBL011w::kanMX4*	
NIU8	*MATα his3 leu2 met15 ura3 YKR067w::kanMX4 YBL011w::HIS3 [pGAL1::GAT1 URA3]*	*gat1 Δgat2Δ+[pGAL1::GAT1 URA3]*
ZAFU1	*MATα his3 leu2 met15 ura3 YKR067w::kanMX4 YBL011w::HIS3 [pGAL1::AtGPAT1 LEU2]*	*gat1 Δgat2Δ+[pGAL1::AtGPAT1 LEU2]*

Table S2. A list of primers used for cloning of individual genes into YEplac181 vector.

Gene	ORF length (bp)	Primer with restriction site	Nucleotide sequence (5'-3')
At1g06520/AtGPAT1	1758	AtGPAT1-FP BamHI	AGCTCGGATCCACTATGGTTTTACCAGAGCTTC
		AtGPAT1-RP SalI	GACTGGTCGACAGTTTATCTGACAATGCCTTC
At1g02390/AtGPAT2	1593	AtGPAT2-FP BamHI	ATCAGGATCCACCATGTCCGGTAATAAGATCTCG
		AtGPAT2-RP SalI	TGCAGTCGACAATTATTTTTTCTTGACAACTCCG
At4g01950/AtGPAT3	1563	AtGPAT3-FP BclI	TGAATGATCAAACATGTCCGCCAAGATTTC
		AtGPAT3-RP SalI	ACAAGTCGACCGAACTCATCACTCACTCATCA
At1g01610/AtGPAT4	1512	AtGPAT4-FP BglII	AGAAAGATCTAAGATGTCTCCGGCGAAGAA
		AtGPAT4-RP SalI	AAAGGTCGACTGGATTATTACTCCATGGACTTGGT
At3g11430/AtGPAT5	1509	AtGPAT5-FP HindIII	TCTCAAGCTTCAAAATGGTTATGGAGCAAGCTGG
		AtGPAT5-RP SalI	AACTGTCGACTTCAATGGAGACAAGGCTCGAAAGT
At2g38110/AtGPAT6	1506	AtGPAT6-FP BamHI	AAGAGGATCCAACATGGGAGCTCAGGAGAAACGG
		AtGPAT6-RP SalI	TGCAGTCGACATCACGTCTTCTCCTTCTTCACC
At5g06090/AtGPAT7	1503	AtGPAT7-FP BglII	AACAAGATCTAACATGGAGTCATCAACTACAACATCGT
		AtGPAT7-RP SalI	GTGTGTCGACTTAATGCAAAAAAGGTTTGAAAGTGGTG
At4g00400/AtGPAT8	1503	AtGPAT8-FP HindIII	AAGAAAGCTTCAAAATGTCTCCGGAGAAGAAGAGTC
		AtGPAT8-RP SalI	TACTAGTCGACTCACTTCTTGGTGTTGTTGATAGAC
GAT1	2232	ZZF1 FP BamHI	GGATCCAACATGTCTGCTCCCGCTGCCGATCAT
		ZZF2 RP XhoI	CTCGAGTCATTCTTTCTTTTCGTGTTCTCT
GAT2	2280	ZZF3 FP BamHI	GGATCCAACATGCCTGCACCAAAACTCACGGAG
		ZZF4 RP XhoI	CTCGAGCTACGCATCTCCTTCTTTCCCTTC

Table S3. A list of primers used for cloning of individual genes into pYES2-Kan-ADH1 vector.

Gene	ORF length (bp)	Primer with restriction site	Nucleotide sequence (5'-3')
At1g06520/AtGPAT1	1758	AtGPAT1-1 FP BamHI	AGCTCGGATCCACTATGGTTTTACCAGAGCTTC
		AtGPAT1-1 RP SalI	GACTGGTCGACAGTTTATCTGACAATGCCTTC
At1g02390/AtGPAT2	1593	AtGPAT2-2-FP Kpn	ATCAGGTACCATGTCCGGTAATAAGATCTCG
		AtGPAT2-2-RP KpnI	TGCAGGTACCAATTATTTTTTCTTGACAACTCCG
At4g01950/AtGPAT3	1563	AtGPAT3-3-FP KpnI	TGAAGGTACCAAACATGTCCGCCAAGATTTC
		AtGPAT3-3-RPKpnI	ACAAGGTACCGAACTCATCACTCACTCATCA
At1g01610/AtGPAT4	1512	AtGPAT4-4-FP KpnI	AGAAGGTACCAAGAAAGATGTCTCCGGCGAAGA
		AtGPAT4-4-RP KpnI	AAAGGGTACCTGGATTATTACTCCATGGACTTGGT
At3g11430/AtGPAT5	1509	AtGPAT5-5-FP KpnI	TCTAGGTACCGAAATGGTTATGGAGCAAGCTGG
		AtGPAT5-5-RP KpnI	AACTGGTACCTTCAATGGAGACAAGGCTCGAAAGT
At2g38110/AtGPAT6	1506	AtGPAT6-6-FP BamHI	AAGAGGATCCAACATGGGAGCTCAGGAGAAACGG
		AtGPAT6-6-RPSalI	TGCAGTCGACATCACGTCTTCTCCTTCTTCACC
At5g06090/AtGPAT7	1503	AtGPAT7-7-FP KpnI	AACAGGTACCATGGAGTCATCAACTACAACATCGT
		AtGPAT7-7-RP KpnI	GTGTGGTACCTTAATGCAAAAAAGGTTTGAAAGTGGTG
At4g00400/AtGPAT8	1503	AtGPAT8-8-FP KpnI	AAGAGGTACCAAAATGTCTCCGGAGAAGAAGAGTC
		AtGPAT8-8-RP KpnI	TACTAGGTACCTCACTTCTTGGTGTTGTTGATAGAC
GAT1	2232	ZZF1 FP BamHI	GGATCCAACATGTCTGCTCCCGCTGCCGATCAT
		ZZF2 RP XhoI	CTCGAGTCATTCTTTCTTTTCGTGTTCTCT
GAT2	2280	ZZF3 FP BamHI	GGATCCAACATGCCTGCACCAAAACTCACGGAG
		ZZF4 RP XhoI	CTCGAGCTACGCATCTCCTTCTTTCCCTTC
At1g32200/ATS1	1380	ZZF144 FP KpnI	CATAGGTACCATGACTCTCACGTTTTCCTCCT
		ZZF145 RP XhoI	GACACTCGAGCTAATTCCAAGGTTGTGACAAAG
SLC1/YDL052c	912	SLC1 FP BamHI	AGTTGGATCCATAATGAGTGTGATAGGTAGGTTCTT
		SLC1 RP BamHI	AACTGGATCCATGTGGTGGTGGCTTAATGCAT
PST1/CST26/YBR042c	1194	lei5-5 FP BamHI	AGCTCGGATCCAACATGCTGCATCAAAAAATAGCTC
		lei5-5 RP XhoI	GACTGCTCGAGTCAAAAAATAAAACAATAAAGTTTATAAAC
At1g75020/LPAT4	1137	LPAT4 FP BglII	GGAAGATCTAACATGGAAGTTTGCGGGGATCT
		LPAT4 RP XhoI	CCGCTCGAGGCATCCCAGAGATATTTTCTGC
At5g13760	1710	myc1-1-FP BamHI	AGGTCGGATCCAACATGGGTGCAACAGAGCCC
		myc 1-1-RP XhoI	GCTTCTCGAGCTAAGCTAGAGCACGGCGAA
At1g25500	1467	myc 2-2-FP BamHI	AGCTCGGATCCAACATGAGTGACTCCACTAACAGCG
		myc 2-2-RP XhoI	GTACGCTCGAGTCAAGCGGTCGTCAAAACTT
YOR161c	1620	lei10-10 FP BamHI	AGCTCGGATCCAACATGCCATTGAATGAAAAATACGA
		lei10-10 RP XhoI	GACTGCTCGAGTCACACATTCTGGTGACTCAATTT
